# Caffeine restricts hepatitis B virus transcription by inhibiting *γ*-H2AX formation

**DOI:** 10.3389/fmicb.2025.1706957

**Published:** 2025-11-10

**Authors:** Fangli Liao, Siyi Sun, Wenjuan Huang, Liping Yang, Qin Hu, Weixian Chen, Chaolin Tu, Linshan Jiang

**Affiliations:** 1 Department of Laboratory Medicine, The Second Affiliated Hospital of Chongqing Medical University, Chongqing, China; 2 Department of Laboratory Medicine, Chongqing Orthopedic Hospital of Traditional Chinese Medicine, Chongqing, China; 3 Department of Laboratory Medicine, Chongqing University Three Gorges Hospital, Chongqing University, Chongqing, China

**Keywords:** caffeine, *γ*-H2AX, core promoter, PPARα, nutritional intervention

## Abstract

The influence of caffeine on human health has been widely studied, but its relevance to hepatitis B virus (HBV) remains unclear. Here, we report that exogenous caffeine suppresses HBV RNA and core protein expression in hepatoma cells. Mechanistically, caffeine reduces the DNA damage marker *γ*-H2AX, which in turn diminishes HBV transcription. Functional assays revealed that *γ*-H2AX enhances HBV core promoter activity by facilitating the recruitment of peroxisome proliferator–activated receptor-*α* (PPARα). Chromatin immunoprecipitation confirmed that reduced *γ*-H2AX levels impair the binding of PPARα to the HBV core promoter. These findings establish a *γ*-H2AX–PPARα signaling axis that promotes HBV transcription and demonstrate that caffeine interferes with this pathway. In summary, our study demonstrated that *γ*-H2AX may serve as a nutritionally targetable node, supporting dietary and adjunct strategies for HBV management.

## 1 Introduction

Chronic hepatitis B virus (HBV) infection is a major public health problem (GBD 2019 Hepatitis B Collaborators, 2022). Despite the widespread availability of an effective prophylactic vaccine, approximately 257 million people are living with HBV infection (Hsu et al., 2023). Therefore, there remains an urgent need to seek potential prevention and treatment strategies. HBV is a member of the hepadnaviridae family of enveloped viruses, with a 3.2 kb double-stranded DNA genome (Hatzakis et al., 2006). Upon infection, the nucleocapsid releases viral DNA, which is transported to the nucleus and transforms into a covalently closed circular DNA (cccDNA) minichromosome. cccDNA serves as the template for transcription of (i) the 3.5-kb pregenomic (pgRNA) and precore (pre-C) RNAs from the core/precore promoter; (ii) the 2.4-kb and 2.1-kb surface mRNAs from the preS1 and preS2/S promoters; and (iii) the 0.7-kb X mRNA initiated from the X promoter (Gomez-Moreno et al., 2024; Karayiannis, 2017). Hence, controlling HBV at the transcription level proves to be an effective means to restrict gene expression of HBV.

Coffee, one of the most popular beverages around the world (van Dam et al., 2020), has been increasingly studied for its potential health benefits. For instance, Hiroroshi et al. reported that the coffee extract at the suitable concentrations exhibits significant antiviral activity and demonstrates the virucidal effects against herpes simplex virus type 1 (Utsunomiya et al., 2008). Another study indicated that pure caffeine significantly inhibits genotype 2a hepatitis C virus (HCV) replication *in vitro* (Batista et al., 2015). Together, these findings support the potential of caffeine as an antiviral agent. Clinical studies have also reported an inverse association between coffee consumption and HBV-associated diseases (Barré et al., 2022). However, little research has been conducted on the anti-HBV mechanisms of caffeine. Thus, further studies are needed to explore the mechanisms of caffeine to regulate HBV activity.

Viruses interact with their host cells to facilitate replication and transcription, which often lead to the activation of DNA damage response (DDR) (Wu et al., 2023; Ning et al., 2023; Studstill et al., 2023; Zhang et al., 2025). Some viruses incorporate their DNA into the genome of their hosts as part of life cycle, which induces a direct DNA break and triggers DDR (Weitzman and Fradet-Turcotte, 2018). Damaged DNA activate ataxia telangiectasia and Rad3 related (ATR), ataxia telangiectasia mutated (ATM) and DNA-dependent protein kinase (DNA-PK). Then, numerous downstream factors are phosphorylated by the PIKKs including H2AX is phosphorylated to form *γ*-H2AX. *γ*-H2AX is a crucial component in DNA damage repair and serves as a biomarker of DDR (Kinner et al., 2008). RNA-seq data have showed that the overexpression of *γ*-H2AX in 19 types of cancers and *γ*-H2AX was positively correlated with the poor survival rate of hepatocellular carcinoma (HCC) patients, indicating the pivotal role of *γ*-H2AX in HCC (Hu et al., 2023). HBV is the major cause of HCC. however, the role of *γ*-H2AX in HBV transcription and replication remains insufficiently characterized. Interestingly, Sarkaria et al. have proved caffeine is an inhibitor of ATR and ATM kinases, which can inhibit *γ*-H2AX formation (Sarkaria et al., 1999). Consequently, we hypothesized that *γ*-H2AX may serve as a key factor underlying the anti-HBV effects of caffeine.

In this study, we examined the ability of caffeine to restrict HBV transcription. Our findings revealed that caffeine inhibits the formation of *γ*-H2AX and further suppresses HBV core promoter activity. We also screened for core promoter-related transcription factors regulated by *γ*-H2AX and identified the transcription factor PPARα as a key factor influenced by caffeine-mediated HBV transcription. Taken together, our findings elucidate a mechanism by which a common dietary component modulates HBV at the transcriptional level and suggest that caffeine may have a beneficial role as a nutrition-linked adjunct in HBV management.

## 2 Materials and methods

### 2.1 Cell culture

The human HCC cell lines HepG2, HepG2.2.15 and Huh7 cells were obtained from the Central Laboratory of the Second Affiliated Hospital, Chongqing Medical University (Chongqing, China). All cell lines were maintained in dulbecco’s modified eagle medium DMEM (Gibco, USA) with 10% fetal bovine serum (Gibco, USA) and 1% penicillin–streptomycin (Beyotime, China). All cells were cultured in a humidified incubator (Thermo, USA) at 37 °C with 5% CO2.

### 2.2 Cell viability assay

Cell Counting Kit-8 (CCK-8) assay (Beyotime, China) was used to assess cytotoxicity of caffeine. Briefly, 5 × 103 cells were seeded in the 96-wells plates, then treated with caffeine at indicated concentrations. After 48 h, 10 μL CCK-8 solution was added to each well and incubated for 1 h at 37 °C. Finally, the absorbance at 450 nm was measured by microplate reader (BioTek Synergy H1, USA).

### 2.3 Immunohistochemical staining

Liver tissue sections from HBV transgenic mice and wild-type mice were obtained from Professor Chen Juan. The sections were deparaffinized, rehydrated and then treated with EDTA antigen repair buffer (pH 9.0) and stained following the Servicebio (Wuhan, China) immunofluorescent staining instruction.

### 2.4 Western blotting

Cells were scraped and then lysed in lysis buffer containing protein inhibitor at 4 °C for 15 min. The concentration of protein was quantified via bicinchoninic acid (BCA) protein assay (Beyotime, China). Total protein was separated by SDS-PAGE and transferred to a polyvinylidene fluoride (PVDF) membrane (GE Healthcare, USA). The membranes were incubated with rabbit anti-H2AX (Proteintech, China), rabbit anti-*γ*-H2AX (Abcam, UK), mouse anti-HBc (Dako, Danmark), and rabbit anti-PPARα (Abcam, UK) and mouse anti-*β*-actin (Boster, China) primary antibodies at 4 °C overnight, and subsequently washed and incubated with secondary antibodies at 37 °C for 1 h. Finally, blots were developed using ECL western blotting reagents (NCM Biotech, China).

### 2.5 RNA extraction and quantitative RT-PCR

Total RNA was extracted using TRIzol reagent (TaKaRa, Japan) and 2 μg RNA was converted to cDNA using the PrimeScript RT reagent kit with gDNA Eraser (TaKaRa, Japan) to generate cDNA. Then, for quantitative PCR, cDNA and primers were mixed with TB GREEN PCR Master Mix. The relative expression levels of each gene were calculated and normalized relative to *β*-actin expression level using the 2-∆∆Ct method. The primer sequences used are as follows: HBV total RNA (F: 5’-ACCGACCTTGAGGCATACTT-3′, R: 5’-GCCTACAGCCTCCTAGTACA-3′); 3.5-kb RNA (F:5’-GCCTTAGAGTCTCCTGAGCA-3′, R:5’-GAGGGAGTTCTTCTTCTAGG-3′); PPARα (F:5’-GCTATCATTACGGAGTCCACG-3′, R:5’-TCGCACTTGTCATACACCAG-3′); β-actin (F:5’-CTCTTCCAGCCTTCCTTCCT-3′, R:5’-AGCACTGTGTTGGCGTACAG-3′).

### 2.6 Dual luciferase activity assay

The luciferase report vectors (pGL3-Cp, pGL3-Xp, pGL3-Sp1, pGL3-Sp2) were constructed and conserved by the Central Laboratory of the Second Affiliated Hospital, Chongqing Medical University (Chongqing, China). pGL3-Cp, pGL3-Xp, pGL3-Sp1, pGL3-Sp2 were co-transfected with pM02-H2AX into HepG2 cells. pRL-TK vector was used to serve as internal control. The cells were harvested after transfection for 48 h and lysed by passive lysis buffer. The dual luciferase activity was analyzed using the dual-luciferase reporter assay kit (Promega, USA). The luciferase activity was determined by GloMax microplate luminometer (Promega, USA).

### 2.7 Co-immunoprecipitation

HepG2 cells were infected with pcDNA3.1-HBx and pM02-H2AX for 48 h. After 48 h, cells were washed 3 times with cold PBS and collected into WB and IP lysis buffer with protease inhibitor (Beyotime, China), then lysed for 30 min at 4 °C. The lysis buffer was then centrifuged. A portion of the supernatant was used as an input sample, and the HBx antibody, *γ*-H2AX antibody and IgG antibody were added to the remaining supernatant, respectively. Next protein G agarose beads were added to the lysate. The immunoprecipitated protein was measured by western blotting.

### 2.8 Chromatin immunoprecipitation (ChIP)

ChIP assays were conducted with ChIP Kit (Beyotime, China). Briefly, the treated cells were cross-linked on plates with 1% formaldehyde at 37 °C for 10 minuities, then glycine buffer (1:10) was added at room temperature for 5 min to quench the crosslinking reaction. Next, the cells were washed twice with PBS and scraped into cold PBS containing protease inhibitors. Cells were resuspended in buffer A, incubated on ice for 10 min. Then cells were centrifuged and resuspended in buffer B with MNase at 37 °C for 20 min. Add 10 μL of 0.5 M EDTA, mix appropriately and place on ice for 1–2 min to terminate the MNase fragmentation reaction. The next step was to shear the DNA by sonication to obtain 200–1,000 bp of DNA fragments. Anti-PPARα antibody or IgG (negative control) were added and mixed with the sample at 4 °C overnight, then Protein A/G Magnetic Beads/Salmon Sperm DNA were added to precipitate the protein recognized by the primary antibody and the corresponding complex. After being purified, the precipitated DNA was amplified using qPCR. The primer sequences for: F: 5’-GTTTAAAGACTGGGAGGAGTTGG-3′, R: 5’-CGCAGACCAATTTATGCCTACAG-3′.

### 2.9 Enzyme-linked immunosorbent assay (ELISA)

The levels of HBsAg and HBeAg were assessed by an enzyme-linked immunosorbent assay (ELISA) (Jiangsu Meibiao Biotechnology, China). To guarantee that the absorbance readings were within the linear range, the cells supernatant was suitably diluted in PBS buffer. Secreted HBsAg and HBeAg were quantified on ELISA plates in accordance with the manufacturer’s protocol.

### 2.10 Statistical analyses

Data were analyzed by GraphPad Prism 9 and bar graphs presented as means ± standard deviation (SD). Statistical analysis was performed using the student’s t-test. *p*-values < 0.05 denoted statistically significant differences. * *p* < 0.05; ** *p* < 0.01; *** *p* < 0.001; **** *p* < 0.0001.

## 3 Results

### 3.1 Caffeine inhibited HBV transcription and decreased the level of *γ*-H2AX

**Figure 1:**
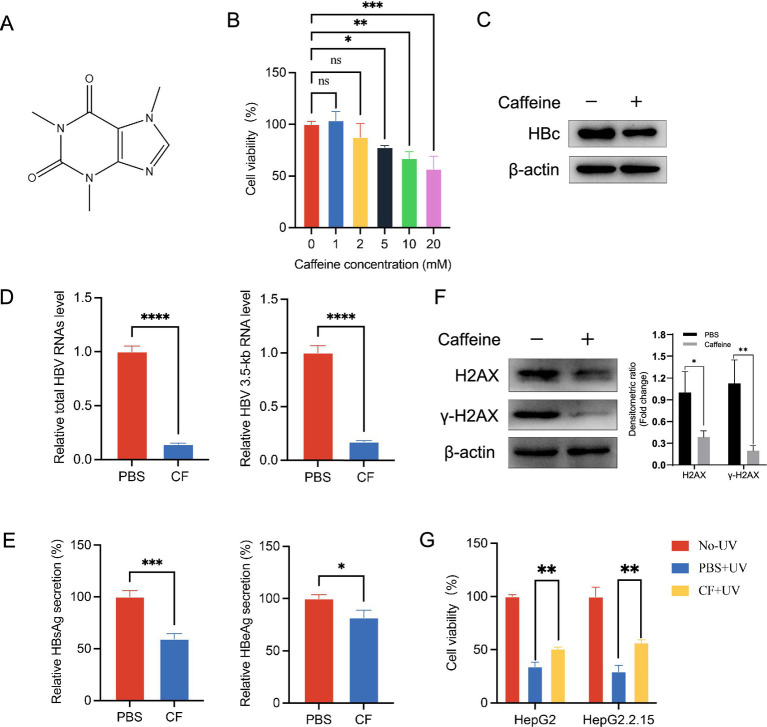
*γ*-H2AX inhibitor caffeine inhibited HBV transcription. **(A)** Chemical structure of caffeine. **(B)** The cell viability of HepG2.2.15 was measured by CCK-8 after treatment with increasing concentrations of caffeine for 48 h. **(C)** The expression of HBc was detected by western blotting 48 h after caffeine treatment. Band intensities were quantified by ImageJ software and normalized to *β*-actin. **(D)** HBV total RNAs and 3.5-kb RNA level were subjected to qRT-PCR 3 days after caffeine treatment. **(E)** Secreted HBsAg and HBeAg were evaluated by ELISA. **(F)** Levels of *γ*-H2AX and H2AX in HepG2.2.15 treated with 2 mM caffeine were detected by western blotting. Densitometric quantification was performed using ImageJ, and protein signals were normalized to β-actin. **(G)** Caffeine reduced the UV-induced phosphorylation of H2AX in HepG2 and HepG2.2.15 cells. Cells were pre-incubated with 2 mM caffeine and exposed to 100 μw/cm^2^ UV light. CF: caffeine.

Caffeine has been associated with various disease modulations. In this study, we investigated its effect on HBV replication using HepG2.2.15 cells, which stably express HBV. The chemical structure of caffeine is shown in Figure 1A. To establish a non-cytotoxic concentration for subsequent experiments, we first assessed the cytotoxicity of caffeine on HepG2.2.15 cells treated for 48 h. As shown in Figure 1B, cell viability remained largely unchanged at 1 mM and 2 mM compared to the 0 mM control. In contrast, treatment with 5 mM, 10 mM, and 20 mM caffeine significantly suppressed cell viability, with the most pronounced effect observed at 10 mM. Consequently, a lower, non-cytotoxic concentration of 2 mM was selected for further analysis. Following 48-h treatment with 2 mM caffeine, western blot analysis revealed a significant reduction in HBc protein expression (Figure 1C). Consistent with this finding, total HBV RNA and 3.5-kb RNA levels were also decreased (Figure 1D). Furthermore, the ELISA results indicated a decrease in the secretion of HBsAg and HBeAg in cells supernatant (Figure 1E). To confirm whether this inhibitory effect also occurs in an infection model, we performed an HBV infection assay using HepG2-NTCP cells, which support authentic viral entry and replication. The experimental procedure is illustrated in Supplementary Figure S1A. After HBV infection, cells were treated with 2 mM caffeine or PBS. As shown in Supplementary Figures S1B,C, caffeine significantly reduced HBV 3.5-kb RNA and total HBV RNA levels at 1–3 days post-infection (dpi) compared with PBS control, indicating that caffeine suppresses HBV transcription during infection. Taken together, the data showed that caffeine could be an inhibitor of HBV transcription.

To elucidate the mechanism by which caffeine suppresses HBV transcription, we considered prior reports that caffeine attenuates the DDR by inhibiting ATR/ATM activity and thereby reducing *γ*-H2AX formation (Block et al., 2004). Thus, we speculated that caffeine inhibits *γ*-H2AX to repress HBV transcription. Consistent with this finding, western blotting analysis revealed that HepG2.2.15 cells with caffeine for 48 h showed a lower level of *γ*-H2AX than that with PBS, which proved caffeine’s effect of repressing *γ*-H2AX in HCC cells (Figure 1F). Moreover, Western blot analysis (Supplementary Figure S1D) revealed that *γ*-H2AX levels progressively increased following HBV infection, coinciding with HBc accumulation, while caffeine treatment markedly attenuated both *γ*-H2AX and HBc expression. To further examine whether caffeine promotes survival following DNA damage in hepatocellular carcinoma cells, we used UV light to induce damage. Hepatoma carcinoma cells HepG2 and HepG2.2.15 were pretreated with 2 mM caffeine for 24 h and then exposed to UV light for 6 h. Then the cell survival was measured by using a CCK-8 assay. As the result shown in Figure 1G, caffeine increased the survival after 100 μw/cm^2^ UV exposure in both HepG2 and HeG2.2.15. Collectively, these data indicate that caffeine reduces H2AX phosphorylation and protects cells from UV-induced death.

### 3.2 *γ*-H2AX promoted HBV transcription

**Figure 2:**
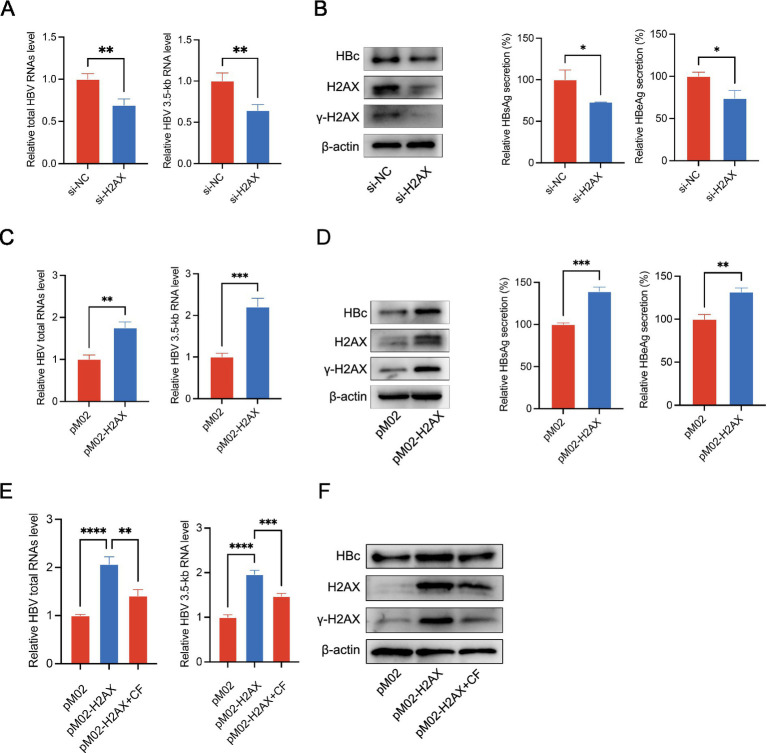
*γ*-H2AX promoted HBV transcription. **(A,B)** H2AX expression was silenced in HepG2.2.15 cells by si-H2AX. **(C,D)** HepG2.2.15 cells were transfected with plasmids pM02-H2AX. **(E,F)** HepG2.2.15 cells were transfected with pM02-H2AX plasmids, then treated with caffeine 24 h later. HBV total RNAs and 3.5-kb RNA level were subjected to qRT-PCR. The expression of HBc and H2AX was detected by western blotting. Secreted HBsAg and HBeAg were evaluated by ELISA.

Based on the above findings, we hypothesized that caffeine suppresses HBV by reducing H2AX phosphorylation. Hence, we investigated whether *γ*-H2AX played a role in the life cycle of HBV. HepG2.2.15 cells were transfected with small-interfering RNA targeting H2AX (si-H2AX) to reduce their ability to express both H2AX and *γ*-H2AX. The knockdown efficiency was verified at protein levels and the cell viability was detected by CCK-8 assay (Supplementary Figures S2, S3). H2AX knockdown reduced total HBV RNA and the 3.5-kb pregenomic RNA (pgRNA), as measured by RT-qPCR (Figure 2A). Consistently, silencing H2AX diminished intracellular HBc and decreased secretion of HBsAg and HBeAg in the culture supernatant (Figure 2B). Conversely, transfection of HepG2.2.15 cells with an H2AX overexpression plasmid (pReceiver-M02-H2AX) or an empty vector control (pReceiver-M02) revealed that H2AX overexpression increased the levels of total HBV RNA and 3.5-kb RNA (Figure 2C), and elevated the HBc protein, as well as HBsAg/HBeAg secretion in the cell supernatant (Figure 2D). Together, these data suggested *γ*-H2AX might have a functional role in the regulation of HBV transcription activity. To test whether caffeine counteracts *γ*-H2AX-driven activation, H2AX-overexpressing HepG2.2.15 cells were treated with caffeine (2 mM). Caffeine abrogated the H2AX-mediated increases in total HBV RNA, pgRNA, and HBc (Figures 2E,F). These results suggested that *γ*-H2AX acts as a positive regulator of HBV transcription.

### 3.3 *γ*-H2AX enhanced core promoter activity by upregulating PPARα

**Figure 3:**
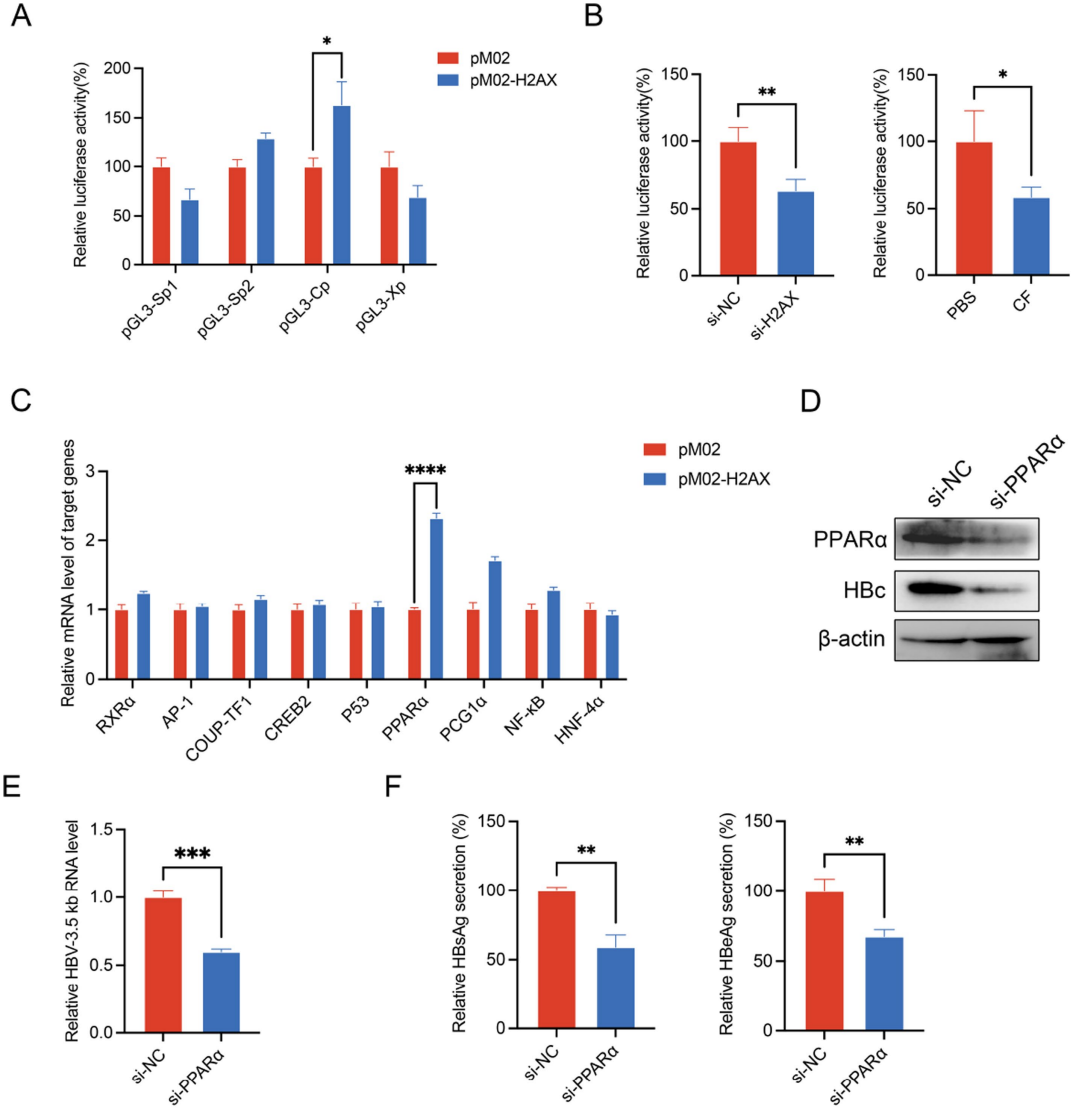
*γ*-H2AX enhanced the activity of HBV core promoter. **(A)** HepG2 cells were transfected with four luciferase reporter plasmids and pM02-H2AX. pRL-TK vector was used to serve as internal control. Luciferase activities were measured at 48 h post-transfection. **(B)** HepG2 cells were transfected with pGL3-Cp after treated with caffeine or transfection of si-H2AX for 24 h. **(C)** The levels of known transcription factors were evaluated by qRT-PCR. HepG2.2.15 cells were transfected with pM02-H2AX. **(D–F)** HepG2.2.15 cells were transfected with si-PPARα. HBV 3.5-kb RNA, HBc, HBsAg and HBeAg were detected by qRT-PCR, western blotting, and ELISA, respectively.

To elucidate the mechanisms through which *γ*-H2AX promotes HBV transcription, we examined the activity of the four HBV promoters (Xp, Sp1, Sp2, and Cp) using a dual-luciferase reporter assay. The results demonstrated that upregulation of *γ*-H2AX specifically enhanced core promoter (Cp) activity (Figure 3A). In contrast, reduction of *γ*-H2AX by caffeine or downregulation of H2AX both significantly suppressed core promoter activity (Figure 3B; Supplementary Figure S4). Given that previous studies have reported the regulation of the HBV core promoter by transcription factors including AP-1, PPARα, RXRα, and CREB (Qu and Brown, 2021), we postulated that *γ*-H2AX might modulate Cp activity by influencing such factors. As shown in Figure 3C, overexpression of H2AX increased the mRNA level of PPARα obviously. We subsequently verified the functional role of PPARα in HBV transcription. PPARα silencing reduced the 3.5-kb pregenomic RNA, HBc, and the secretion of HBsAg and HBeAg (Figures 3D–F). Collectively, these results indicate that *γ*-H2AX enhances HBV core promoter activity, at least in part, by upregulating PPARα.

### 3.4 *γ*-H2AX and PPARα involved in caffeine-mediated HBV transcriptional repression

**Figure 4:**
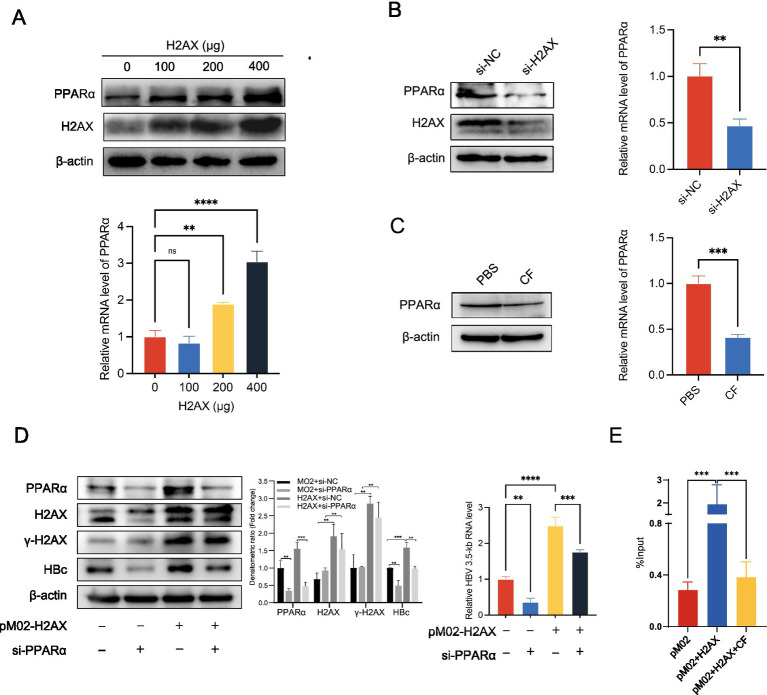
The impact of *γ*-H2AX on PPARα. **(A)** HepG2.2.15 cells were transfected H2AX protein plasmids at 0, 100, 200, 400 μg, respectively. Western blotting and qRT-PCR determined levels of PPARα protein and mRNA. **(B,C)** Western blotting and qRT-PCR determined levels of PPARα protein and mRNA in HepG2.2.15 cells transfected with si-H2AX or treated with 2 mM caffeine. **(D)** HepG2.2.15 cells were transfected with plasmids pM02-H2AX 24 h after transfection of si-PPARα. The HBV 3.5-kb RNA level was detected by qRT-PCR, and the HBc level was determined by western blotting. Densitometric quantification of protein signals was normalized to β-actin. **(E)** ChIP-quantitative PCR was performed to measure the binding of PPARα at the HBV core promoter in HepG2.2.15 cells transfected with plasmids pM02-H2AX and treated with caffeine.

To confirm the relationship between *γ*-H2AX and PPARα, we checked the change of protein and mRNA level of PPARα in HepG2.2.15 upon H2AX overexpression. We transfected H2AX protein plasmids at 0, 100, 200, 400 μg respectively, and the expression of H2AX and PPARα was assessed. As shown in Figure 4A, we found that the levels of PPARα protein and mRNA were upregulated by H2AX in a dose-dependent manner. In addition, a marked reduction in both protein and mRNA level of PPARα were observed in cells with H2AX knockdown or caffeine treatment (Figures 4B,C). Collectively, the above results suggested that *γ*-H2AX might upregulate PPARα to enhance the activity of HBV core promoter. To confirm the relationship between caffeine, *γ*-H2AX, PPARα, and activity of HBV core promoter, we conducted rescue experiments. Knockdown of PPARα abrogated the *γ*-H2AX-induced increase in HBV 3.5-kb RNA and HBc protein levels, as determined by qRT-PCR and western blotting (Figure 4D), indicating that *γ*-H2AX enhances HBV transcription by upregulating PPARα.

We further performed ChIP assay to evaluate the interaction between PPARα and HBV core promoter in the case of increased or decreased *γ*-H2AX by overexpression of H2AX or caffeine treatment. Chromatin was extracted from the treated cells and immunoprecipitated using an anti-PPARα antibody; the bound DNA was then quantified by RT-PCR. As shown in Figure 4E, elevated *γ*-H2AX levels enhanced the binding of PPARα to the core promoter. Importantly, caffeine treatment decreased the enhancement of PPARα recruitment to the HBV core promoter induced by *γ*-H2AX, indicating that *γ*-H2AX mediates this process. These results demonstrate that *γ*-H2AX enhances core promoter activity by facilitating the association between PPARα and the promoter.

### 3.5 HBx played a role in HBV-mediated upregulation of *γ*-H2AX

**Figure 5:**
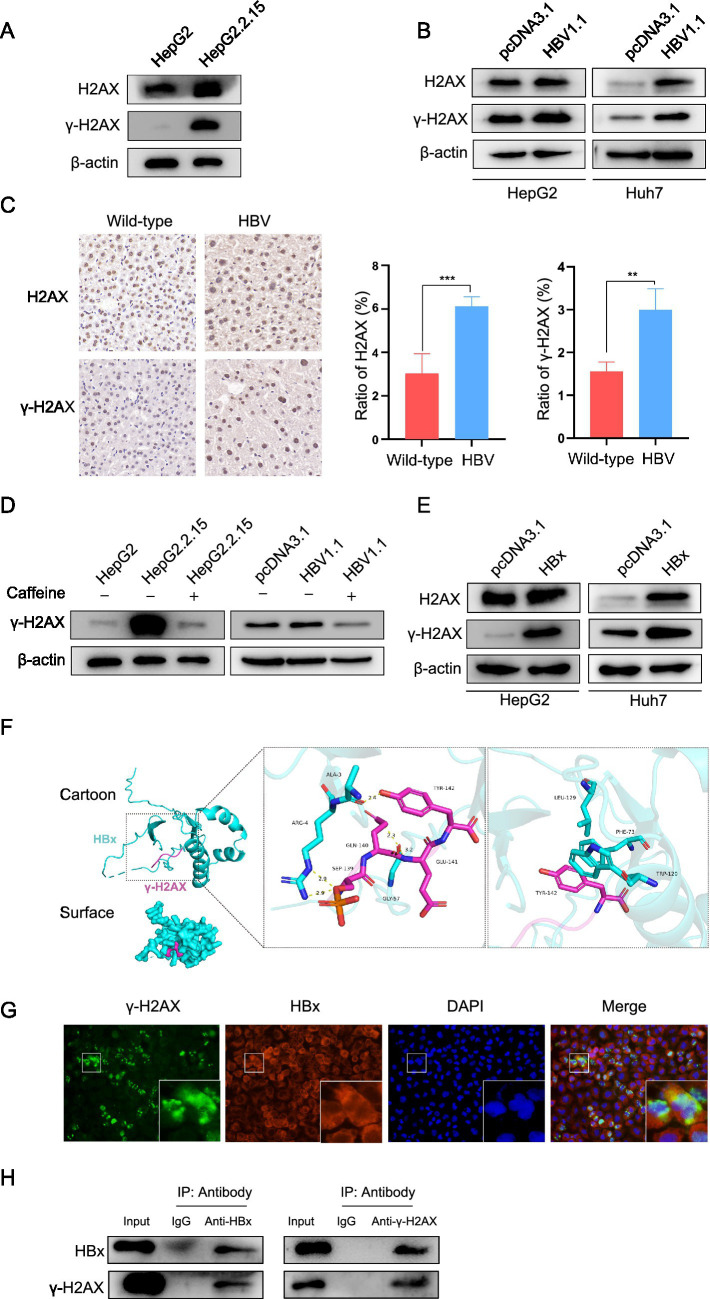
HBx played a role in HBV-mediated upregulation of *γ*-H2AX. **(A)** The level of H2AX and *γ*-H2AX was detected by western blotting in HepG2 and HepG2.2.15 cells. **(B)** The HepG2 and Huh7 cells were transfected with pcDNA3.1-HBV1.1, H2AX and *γ*-H2AX expression was detected by western blotting. **(C)** Immunohistochemical detection of H2AX and *γ*-H2AX in liver tissue sections from wild-type and HBV transgenic mice. **(D)** HBV stable expressing cells and transient expressing cells were treated with caffeine. *γ*-H2AX was analyzed by western blotting. **(E)** The pcDNA3.1-HBx constructs were transfected to HepG2 and Huh7 cells, H2AX and *γ*-H2AX was analyzed by western blotting. **(F)** The protein–protein molecular docking technique predicted that *γ*-H2AX and HBx had 5 bonding hydrogen bonds and 3 hydrophobic interactions. **(G)** IF assay showing the localization of *γ*-H2AX and HBx in HuH-7 cells transfected with HBx overexpression plasmids. **(H)** Co-ip confirmed the interaction between HBx and *γ*-H2AX. HBx and *γ*-H2AX were analyzed by western blotting. Input: total protein. IgG: negative control. IP: total protein incubated with anti-HBx or anti-*γ*-H2AX.

Previous studies have reported that integrated HBV DNA can cause host DNA damage and induce the DDR (Gómez-Moreno and Garaigorta, 2017; Kostyusheva et al., 2019). We ascertained it by measuring the level of H2AX and *γ*-H2AX in HepG2 and HepG2.2.15. It showed that HBV-positive cells exhibited a much higher level of *γ*-H2AX than HBV-negative cells (Figure 5A). The same results were found in HepG2 and HBV transient expressing cells HepG2-HBV1.1 cells, as well as in Huh7 and Huh7-HBV1.1 cells (Figure 5B). Furthermore, immunohistochemical analysis demonstrated significantly higher levels of *γ*-H2AX in HBV transgenic mice compared to wild-type mice (Figure 5C). And as mentioned above, caffeine effectively attenuated the upregulation of *γ*-H2AX induced by HBV (Figure 5D). Given reports that viral genomes are not the only triggers for DDR activation (Gómez-Moreno and Garaigorta, 2017), we tried to investigate other factors involved in the process. The HBV X protein (HBx), which is essential for viral replication, has been shown to interfere with cellular DNA repair mechanisms (Kim et al., 2015; Kim et al., 2022; Wang et al., 1994; Qadri et al., 2011). Moreover, it has reported that HBx is one of the major factors causing DNA double-strand breaks (DSBs) in HBV-related liver cells (Kim et al., 2015). Thus, we hypothesized there is a connection between HBx and *γ*-H2AX. To test this, we first examined the effect of HBx overexpression on *γ*-H2AX levels and observed a notable increase in both HepG2 and Huh7 cells (Figure 5E). To explore whether this effect might involve direct molecular interaction, we performed computational molecular docking simulations. The results suggested that Lamin A/C and HBx interact through multiple amino acid residues, which is primarily facilitated by hydrogen bonding and hydrophobic interactions (Figure 5F). To experimentally verify this predicted interaction, we next used immunofluorescence and co-immunoprecipitation assays to examine the interaction between HBx and *γ*-H2AX in Huh7 cells (Figures 5G,H). Collectively, these data indicate that HBx contributes to *γ*-H2AX upregulation in HBV infection, likely through a direct binding mechanism, thereby elucidating a novel aspect of HBV-induced DNA damage response.

## 4 Discussion

In this study, we demonstrated caffeine restricted HBV transcription by inhibiting *γ*-H2AX formation, which restricted PPARα expression and then weakened the activity of core promoter. These data position *γ*-H2AX as a nutritionally targetable regulator of HBV transcription and suggest that habitual dietary constituents may influence virological outcomes in chronic hepatitis B.

*γ*-H2AX, the biomarker of DDR, is essential in DNA repair process (Kinner et al., 2008; Luczak and Zhitkovich, 2018; Pan et al., 2011). Apart from its function in DDR, *γ*-H2AX also played a crucial part in the viral life cycle. It has been reported that inhibition of *γ*-H2AX could restrain both replication and production of EVA71 (Yu et al., 2022). Consistent with this finding, our investigation verified that inhibiting *γ*-H2AX could impede the transcription of HBV. Moreover, we observed that HBx could interact with *γ*-H2AX and promote its accumulation. These results indicate that HBV infection triggers a DDR characterized by *γ*-H2AX formation, which may create a favorable environment for viral transcription and replication.

Caffeine has been shown to modulate DDR pathways by acting as an inhibitor of the serine/threonine kinases ATM and ATR, thereby reducing the phosphorylation of H2AX (Block et al., 2004). This biochemical property underlies its reported antitumor and antiviral effects in several DNA and RNA viruses, including HPV, HIV, and HCV (Batista et al., 2015; Kanginakudru et al., 2022; Nunnari et al., 2005). Our present study demonstrates that caffeine treatment diminished *γ*-H2AX levels in hepatoma cells and increased cell survival under UV-induced DNA damage, consistent with DDR attenuation. These observations corroborate prior reports that caffeine suppresses DNA damage-induced signaling and align with its role as a DDR modulator in cancer and viral systems.

A study from ANRS CO22 Hepather cohort have revealed that coffee intake was associated with the risk of liver fibrosis in chronic HBV patients (Barré et al., 2022). Furthermore, meta-analyses and case–control studies consistently report a dose-dependent reduction in hepatocellular carcinoma risk among HBV carriers who consume coffee (Leung et al., 2011; Ma et al., 2015). At the molecular level, caffeine has been shown to specifically suppress PGE2 synthesis in HBx-positive hepatocytes via the PPAR*γ*-EGR1 pathway (Ong et al., 2011). Consistent with these proposed findings, we found that under the administration of caffeine, the protein level of HBc and the secretion of HBsAg and HBeAg decreased significantly in HBV stable expressing cells. Caffeine treatment also diminished HBV core promoter activity and reduced HBV RNA level. In our study, we confirmed the anti-HBV effect of caffeine and investigated the possible mechanisms.

Building on this mechanistic insight, we identified *γ*-H2AX as a critical mediator linking DDR and HBV transcriptional regulation. It is commonly known that 3.5-kb RNA production requires effective transcription of the HBV core promoter. Notably, silencing PPARα largely abolished the *γ*-H2AX – induced increases in HBV RNA and HBc protein levels, demonstrating that the transcriptional enhancement mediated by *γ*-H2AX requires the presence and activity of PPARα. This finding provides functional evidence that PPARα acts as a downstream effector of *γ*-H2AX in promoting HBV transcription. Consistently, the ChIP assay demonstrated that *γ*-H2AX could enhance the activity of the core promoter by upregulating PPARα. Importantly, caffeine treatment abrogated this *γ*-H2AX–PPARα axis, leading to decreased HBV RNA, HBc protein, and HBsAg/HBeAg secretion. A previous study reported that ATM/ATR signaling contributes to HBV replication and transcriptional regulation (Brezgin et al., 2019), and caffeine is a known inhibitor of the upstream DDR kinases ATM and ATR (Block et al., 2004). So, it is plausible that caffeine represses HBV transcription by attenuating upstream DDR kinase activity, thereby preventing *γ*-H2AX formation and subsequent PPARα-mediated transcriptional activation. These findings position *γ*-H2AX as a targetable regulator of HBV transcription and suggest that habitual dietary constituents, such as caffeine, may modulate virological outcomes in chronic hepatitis B.

Nevertheless, several limitations of this study should be acknowledged. To achieve a more pronounced experimental effect *in vitro*, we selected a caffeine concentration of 2 mM, which is higher than the physiological levels achievable through normal coffee intake (in the tens of micromolar range). Consistent with our approach, other studies have also employed 2 mM caffeine when investigating its cellular and pharmacological effects (Batista et al., 2015). Our additional data show that caffeine suppresses HBV 3.5-kb RNA expression in a dose-dependent manner (0–2 mM), and 2 mM did not exert cytotoxic effects on HepG2.2.15 cells, supporting the validity of this concentration for mechanistic exploration (Supplementary Figure S5; Figure 1B). This concentration allowed us to clearly demonstrate the inhibitory action of caffeine on HBV transcription, although it may not fully reflect physiological exposure conditions. Moreover, while our data support that caffeine represses HBV transcription through suppression of *γ*-H2AX formation, the precise molecular intermediates linking caffeine, DDR kinases, and HBV replication remain to be elucidated. Further investigations using phosphomimetic H2AX mutants, kinase inhibitors, and *in vivo* caffeine exposure models will be necessary to clarify the detailed signaling cascade and to determine whether *γ*-H2AX acts as the key mediator or as part of a broader DDR regulatory network influencing HBV transcription.

## 5 Conclusion

In summary, we clarified identifies a nutrition-relevant antiviral mechanism wherein caffeine suppresses HBV transcription by limiting *γ*-H2AX formation and downstream PPARα-dependent activation of the core promoter. These findings nominate *γ*-H2AX as a nutritionally targetable node in HBV and provide a testable rationale for integrating dietary considerations into comprehensive HBV management strategies.

## Data Availability

The raw data supporting the conclusions of this article will be made available by the authors, without undue reservation.

## References

[ref1] Barré T. Fontaine H. Ramier C. Di Beo V. Pol S. Carrieri P. . (2022). Elevated coffee consumption is associated with a lower risk of elevated liver fibrosis biomarkers in patients treated for chronic hepatitis B (ANRS CO22 Hepather cohort). Clin. Nutr. 41, 610–619. doi: 10.1016/j.clnu.2022.01.016, PMID: 35124468

[ref2] Batista M. N. Carneiro B. M. Braga A. C. Rahal P. (2015). Caffeine inhibits hepatitis C virus replication in vitro. Arch. Virol. 160, 399–407. doi: 10.1007/s00705-014-2302-1, PMID: 25491197

[ref3] Block W. D. Merkle D. Meek K. Lees-Miller S. P. (2004). Selective inhibition of the DNA-dependent protein kinase (DNA-PK) by the radiosensitizing agent caffeine. Nucleic Acids Res. 32, 1967–1972. doi: 10.1093/nar/gkh508, PMID: 15060176 PMC390360

[ref4] Brezgin S. Kostyusheva A. Bayurova E. Gordeychuk I. Isaguliants M. Goptar I. . (2019). Replenishment of hepatitis B virus cccDNA Pool is restricted by baseline expression of host restriction factors in vitro. Microorganisms 7:110533. doi: 10.3390/microorganisms7110533, PMID: 31698767 PMC6920784

[ref5] GBD 2019 Hepatitis B Collaborators (2022). Global, regional, and national burden of hepatitis B, 1990-2019: a systematic analysis for the global burden of disease study 2019. Lancet Gastroenterol. Hepatol. 7, 796–829. doi: 10.1016/s2468-1253(22)00124-8 35738290 PMC9349325

[ref6] Gómez-Moreno A. Garaigorta U. (2017). Hepatitis B virus and DNA damage response: interactions and consequences for the infection. Viruses 9:10.3390/v9100304. doi: 10.3390/v9100304, PMID: 29048354 PMC5691655

[ref7] Gomez-Moreno A. Guo J. Temple H. M. Ploss A. (2024). Formation and transcriptional regulation of hepatitis B virus covalently closed circular DNA. J. Hepatol. 81, 367–369. doi: 10.1016/j.jhep.2024.03.008, PMID: 38782610

[ref8] Hatzakis A. Magiorkinis E. Haida C. (2006). HBV virological assessment. J. Hepatol. 44, S71–S76. doi: 10.1016/j.jhep.2005.11.017, PMID: 16343681

[ref9] Hsu Y. C. Huang D. Q. Nguyen M. H. (2023). Global burden of hepatitis B virus: current status, missed opportunities and a call for action. Nat. Rev. Gastroenterol. Hepatol. 20, 524–537. doi: 10.1038/s41575-023-00760-9, PMID: 37024566

[ref10] Hu H. Zhong T. Jiang S. (2023). H2AFX might be a prognostic biomarker for hepatocellular carcinoma. Cancer Rep. 6:e1684. doi: 10.1002/cnr2.1684, PMID: 35903980 PMC9875689

[ref11] Kanginakudru S. Gilson T. Jose L. Androphy E. J. (2022). Effects of caffeine, a DNA damage response inhibitor, on papillomavirus genome replication. Pathogens 11:1298. doi: 10.3390/pathogens11111298, PMID: 36365049 PMC9698569

[ref12] Karayiannis P. (2017). Hepatitis B virus: virology, molecular biology, life cycle and intrahepatic spread. Hepatol. Int. 11, 500–508. doi: 10.1007/s12072-017-9829-7, PMID: 29098564

[ref13] Kim S. Lee H. S. Ji J. H. Cho M. Y. Yoo Y. S. Park Y. Y. . (2015). Hepatitis B virus X protein activates the ATM-Chk2 pathway and delays cell cycle progression. J. Gen. Virol. 96, 2242–2251. doi: 10.1099/vir.0.000150, PMID: 25872745

[ref14] Kim E. S. Zhou J. Zhang H. Marchetti A. van de Klundert M. Cai D. . (2022). Hepatitis B virus X protein counteracts high mobility group box 1 protein-mediated epigenetic silencing of covalently closed circular DNA. PLoS Pathog. 18:e1010576. doi: 10.1371/journal.ppat.1010576, PMID: 35679251 PMC9182688

[ref15] Kinner A. Wu W. Staudt C. Iliakis G. (2008). Gamma-H2AX in recognition and signaling of DNA double-strand breaks in the context of chromatin. Nucleic Acids Res. 36, 5678–5694. doi: 10.1093/nar/gkn550, PMID: 18772227 PMC2553572

[ref16] Kostyusheva A. Brezgin S. Bayurova E. Gordeychuk I. Isaguliants M. Goptar I. . (2019). ATM and ATR expression potentiates HBV replication and contributes to reactivation of HBV infection upon DNA damage. Viruses 11:10.3390/v11110997. doi: 10.3390/v11110997, PMID: 31683589 PMC6893526

[ref17] Leung W. W. Ho S. C. Chan H. L. Wong V. Yeo W. Mok T. S. (2011). Moderate coffee consumption reduces the risk of hepatocellular carcinoma in hepatitis B chronic carriers: a case-control study. J. Epidemiol. Community Health 65, 556–558. doi: 10.1136/jech.2009.104125, PMID: 20693491

[ref18] Luczak M. W. Zhitkovich A. (2018). Monoubiquitinated γ-H2AX: abundant product and specific biomarker for non-apoptotic DNA double-strand breaks. Toxicol. Appl. Pharmacol. 355, 238–246. doi: 10.1016/j.taap.2018.07.007, PMID: 30006243 PMC6754567

[ref19] Ma Y. Wang X. Tang N. (2015). Downregulation of mPGES-1 expression via EGR1 plays an important role in inhibition of caffeine on PGE2 synthesis of HBx(+) hepatocytes. Mediat. Inflamm. 2015:372750. doi: 10.1155/2015/372750, PMID: 26538827 PMC4619973

[ref20] Ning K. Kuz C. A. Cheng F. Feng Z. Yan Z. Qiu J. (2023). Adeno-associated virus monoinfection induces a DNA damage response and DNA repair that contributes to viral DNA replication. MBio 14:e0352822. doi: 10.1128/mbio.03528-22, PMID: 36719192 PMC9973366

[ref21] Nunnari G. Argyris E. Fang J. Mehlman K. E. Pomerantz R. J. Daniel R. (2005). Inhibition of HIV-1 replication by caffeine and caffeine-related methylxanthines. Virology 335, 177–184. doi: 10.1016/j.virol.2005.02.015, PMID: 15840517

[ref22] Ong A. Wong V. W. Wong G. L. Chan H. L. (2011). The effect of caffeine and alcohol consumption on liver fibrosis - a study of 1045 Asian hepatitis B patients using transient elastography. Liver Int. 31, 1047–1053. doi: 10.1111/j.1478-3231.2011.02555.x, PMID: 21733095

[ref23] Pan M.-R. Peng G. Hung W.-C. Lin S.-Y. (2011). Monoubiquitination of H2AX protein regulates DNA damage response Signaling. J. Biol. Chem. 286, 28599–28607. doi: 10.1074/jbc.M111.256297, PMID: 21676867 PMC3151101

[ref24] Qadri I. Fatima K. Abde L. H. H. (2011). Hepatitis B virus X protein impedes the DNA repair via its association with transcription factor, TFIIH. BMC Microbiol. 11:48. doi: 10.1186/1471-2180-11-48 21375739 PMC3060106

[ref25] Qu B. Brown R. J. P. (2021). Strategies to inhibit hepatitis B virus at the transcript level. Viruses 13:10.3390/v13071327. doi: 10.3390/v13071327, PMID: 34372533 PMC8310268

[ref26] Sarkaria J. N. Busby E. C. Tibbetts R. S. Roos P. Taya Y. Karnitz L. M. . (1999). Inhibition of ATM and ATR kinase activities by the radiosensitizing agent, caffeine. Cancer Res. 59, 4375–4382. 10485486

[ref27] Studstill C. J. Mac M. Moody C. A. (2023). Interplay between the DNA damage response and the life cycle of DNA tumor viruses. Tumour Virus Res. 16:200272. doi: 10.1016/j.tvr.2023.200272, PMID: 37918513 PMC10685005

[ref28] Utsunomiya H. Ichinose M. Uozaki M. Tsujimoto K. Yamasaki H. Koyama A. H. (2008). Antiviral activities of coffee extracts in vitro. Food Chem. Toxicol. 46, 1919–1924. doi: 10.1016/j.fct.2008.01.031, PMID: 18314244

[ref29] van Dam R. M. Hu F. B. Willett W. C. (2020). Coffee, caffeine, and health. N. Engl. J. Med. 383, 369–378. doi: 10.1056/NEJMra1816604, PMID: 32706535

[ref30] Wang X. W. Forrester K. Yeh H. Feitelson M. A. Gu J. R. Harris C. C. (1994). Hepatitis B virus X protein inhibits p53 sequence-specific DNA binding, transcriptional activity, and association with transcription factor ERCC3. Proc. Natl. Acad. Sci. USA 91, 2230–2234. doi: 10.1073/pnas.91.6.2230, PMID: 8134379 PMC43344

[ref31] Weitzman M. D. Fradet-Turcotte A. (2018). Virus DNA replication and the host DNA damage response. Annu. Rev. Virol. 5, 141–164. doi: 10.1146/annurev-virology-092917-043534, PMID: 29996066 PMC6462412

[ref32] Wu X. Zhou X. Wang S. Mao G. (2023). DNA damage response (DDR): a link between cellular senescence and human cytomegalovirus. Virol. J. 20:250. doi: 10.1186/s12985-023-02203-y, PMID: 37915066 PMC10621139

[ref33] Yu J. Zhang W. Huo W. Meng X. Zhong T. Su Y. . (2022). Regulation of host factor γ-H2AX level and location by enterovirus A71 for viral replication. Virulence 13, 241–257. doi: 10.1080/21505594.2022.2028482, PMID: 35067196 PMC8786350

[ref34] Zhang D. Hou Y. Qiu X. Qu Y. Sun Y. Song C. . (2025). Newcastle disease virus exploits the phospholipid flippase ATP11c–CDC50A complex to promote viral infection. J. Biol. Chem. 301:584. doi: 10.1016/j.jbc.2025.110584 PMC12446626 40812423

