# Supplementary material for: Caffeine restricts hepatitis B virus transcription by inhibiting γ-H2AX formation
Source: Front Microbiol. 2025 Nov 10;16:1706957. doi: 10.3389/fmicb.2025.1706957 (PMC12640983; doi:10.3389/fmicb.2025.1706957)

**Figure S1. Caffeine inhibited HBV transcription and decreased the level of γ-H2AX**


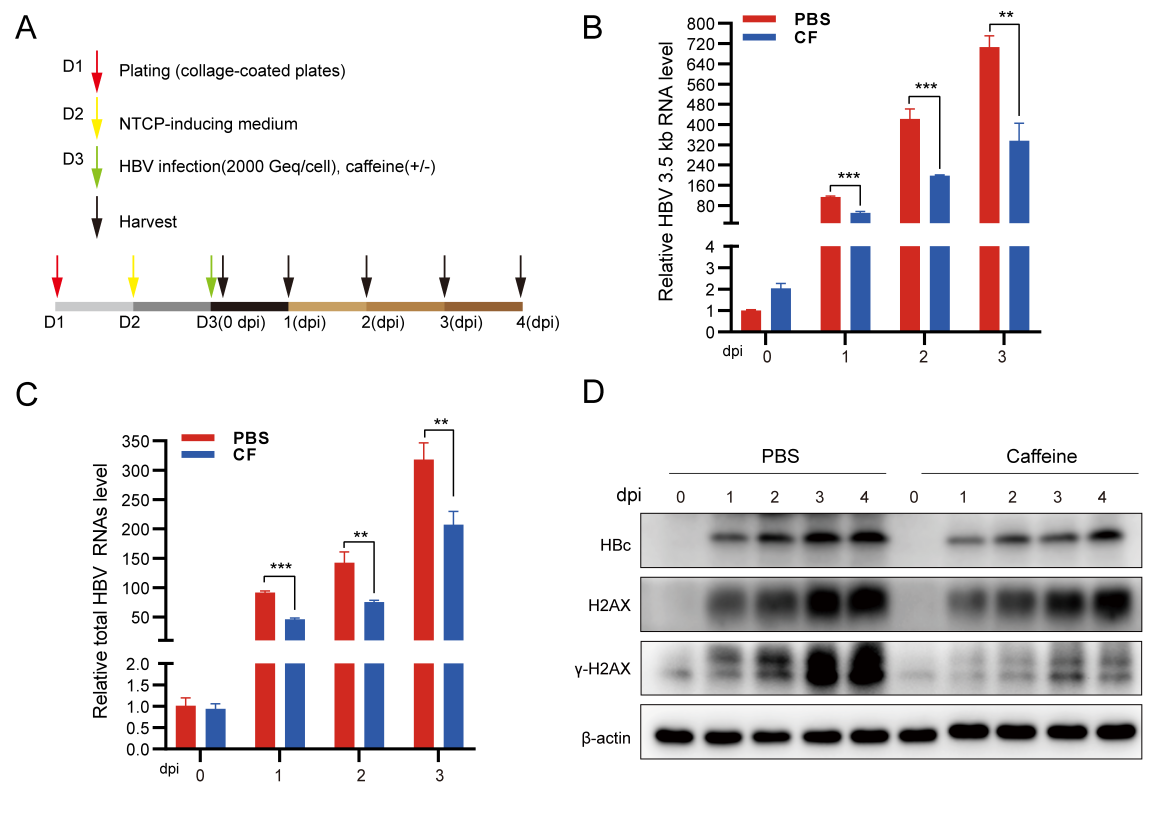


Figure S1. Caffeine inhibited HBV transcription and decreased the level of γ-H2AX. Cells were plated on collagen-coated dishes, cultured in NTCP-inducing medium, and then infected with HBV (2000 Geq/cell) in the presence or absence of 2 mM caffeine. (A) Schematic diagram of the HBV infection procedure in HepG2-NTCP cells.(B)-(C) RT-qPCR detection of 3.5-kb RNA and total HBV RNA. (D) The expression of HBc was detected by western blotting

**Figure S2. The knockdown efficiency of si-H2AX at protein level**


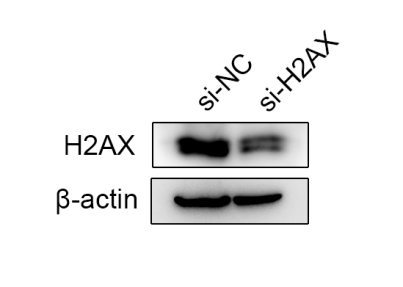


**Figure S3. The cell viability of caffeine**

**
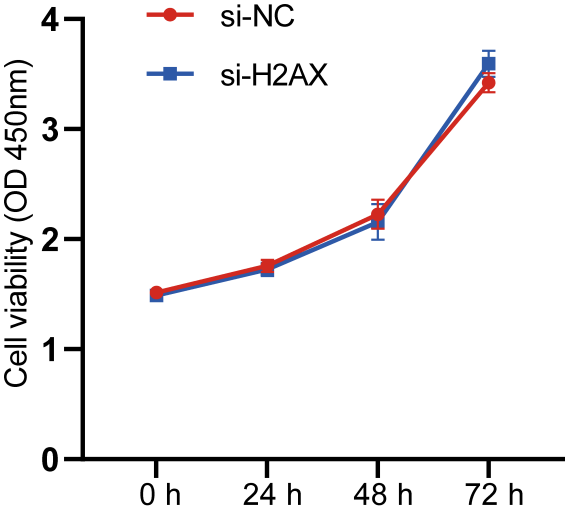
**

**Figure S4. Caffeine reduced the activity of HBV core promoter**


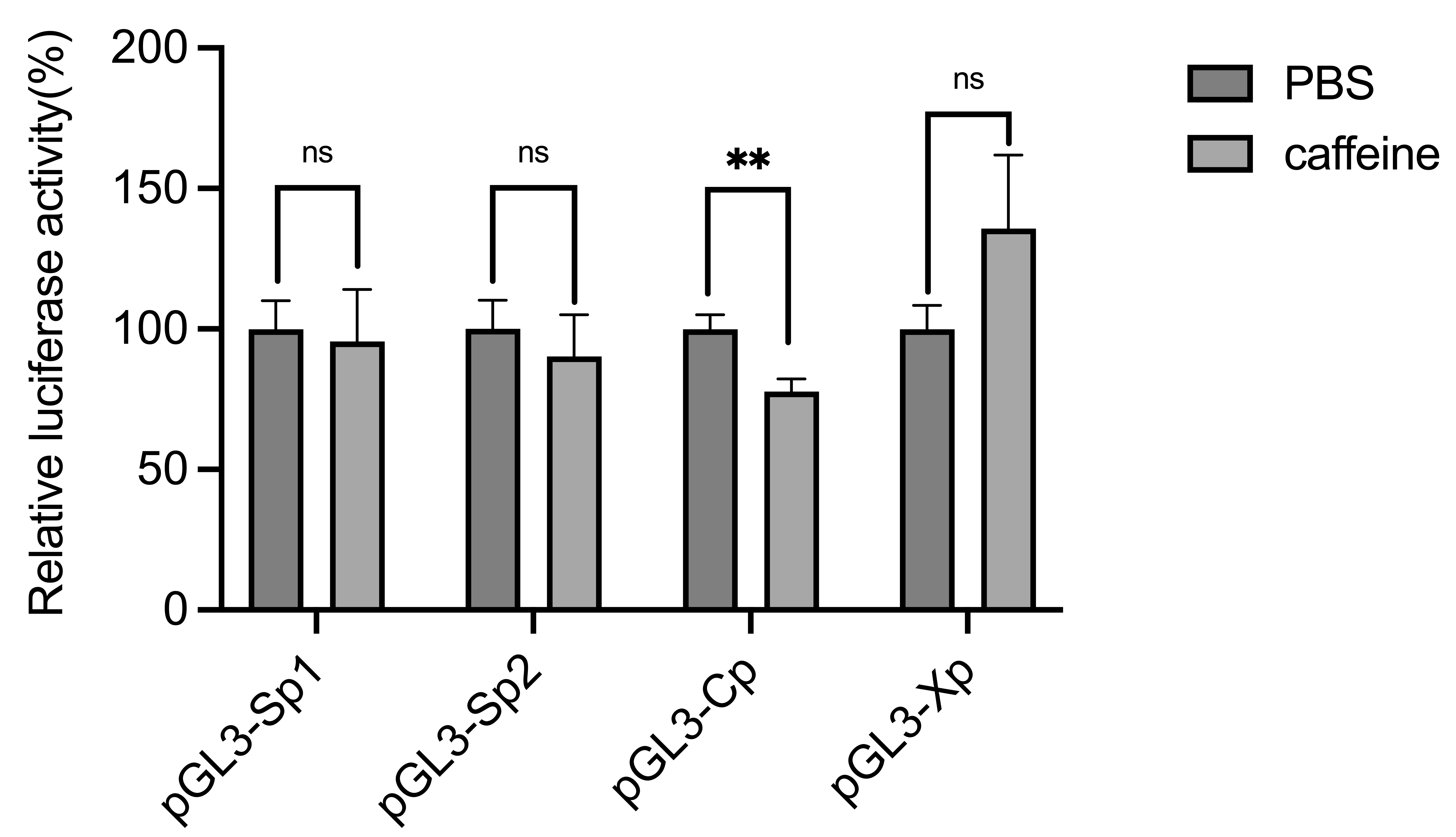


**Figure S5. HBV 3.5 kb RNA were detected by RT-PCR**


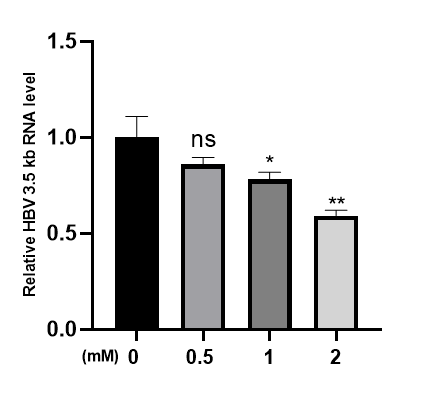

Supplement: Supplementary file 1 [file Data_Sheet_1.docx]
